# Factors Influencing Patient Compliance during Clear Aligner Therapy: A Retrospective Cohort Study

**DOI:** 10.3390/jcm10143103

**Published:** 2021-07-14

**Authors:** Lan Huong Timm, Gasser Farrag, Martin Baxmann, Falk Schwendicke

**Affiliations:** 1 Sunshine Smile, Windscheidstraße 18, 10627 Berlin, Germany; gassertarekf@gmail.com; 2 Orthodentix, Arnoldstrasse 13 b, 47906 Kempen, Germany; martin.baxmann@orthodentix.de; 3 Department of Oral Diagnostics, Digital Health and Health Services Research, Charité—Universitätsmedizin Berlin, Aßmannshauser Straße 4-6, 14197 Berlin, Germany; falk.schwendicke@charite.de

**Keywords:** orthodontics, corrective orthodontics, removable orthodontic appliance, clear aligners, malocclusion, remote consultation, telemedicine, teledentistry, teleorthodontics, distance counseling

## Abstract

Compliance is highly relevant during clear aligner therapy (CAT). In this retrospective cohort study, we assessed compliance and associated covariates in a large cohort of CAT patients. A comprehensive sample of 2644 patients (75.0% females, 25.0% males, age range 18–64 years, median 27 years), all receiving CAT with PlusDental (Berlin, Germany) finished in 2019, was analyzed. Covariates included demographic ones (age, gender) as well as self-reported questionnaire-obtained ones (satisfaction with ones’ smile prior treatment, the experience of previous orthodontic therapy). The primary outcome was compliance: Based on patients’ consistent use of the mobile application for self-report and aligner wear time of ≥22 h, patients were classified as fully compliant, fairly compliant, or poorly compliant. Chi-square test was used to compare compliance in different subgroups. A total of 953/2644 (36.0%) of patients showed full compliance, 1012/2644 (38.3%) fair compliance, and 679/2644 (25.7%) poor compliance. Males were significantly more compliant than females (*p* = 0.000014), as were patients without previous orthodontic treatment (*p* = 0.023). Age and self-perceived satisfaction with ones’ smile prior to treatment were not sufficiently associated with compliance (*p* > 0.05). Our findings could be used to guide practitioners towards limitedly compliant individuals, allowing early intervention.

## 1. Introduction

Technological advancements in computers, mobile phones, internet security, telecommunications, and software allow increased options for networking, information sharing, and consultation in medicine, facilitating remote and cost-effective (tele-)healthcare [1,2,3,4,5,6]. In dentistry, non-contact communication between patients and dentists has been used for various steps along the clinical workflow including initial diagnosis, joint treatment planning, follow-up, and intermediate consultations [1,2,3,4,5,6]. 

The clear aligner technology (CAT) builds on clear thermoformed plastic aligners to correct mild to moderately complex forms of malocclusion [7,8] and has gained popularity in the past years especially for adult orthodontics [9,10]. Although there are a lot of similarities between different CAT systems, they differ in their range of application, methods of construction, aligner thickness, the use of bonded resin attachments, the treatment sequence, and the application duration per aligner. While rapid technological advances lead to a highly paced evolution of these different systems [7,8], there is often limited evidence supporting them [7,8,11,12].

Like other orthodontic treatment appliances, CAT moves teeth by applying compressive and tensile forces to the periodontium. Optimal orthodontic tooth movement occurs when continuous forces are applied and maintained, while given that teeth are moved, the initial forces exerted are higher than those later during each aligner step. Hence, regular change of aligners is needed. In more recent concepts these changes are suggested to be required after one to two weeks. Regularly changing aligners requires a high level of patient compliance [13,14]. Such compliance is further needed as they need to be worn near-permanently (minimum 22 h per day) [15]. 

Compliance for orthodontic therapy has been found to vary between males and females [16] as well as between age groups [17], while overall data on compliance and treatment outcomes in orthodontics are limited and ambiguous [18]. In patients with low compliance, treatment times increase, and the outcome may be compromised [16,19]. 

The present study aimed to evaluate the compliance of aligner patients during remote treatment monitoring and to assess if compliance was associated with demographic or other covariates (e.g., previous orthodontic treatment experience, satisfaction with their current smile). Having knowledge on determinants or indicators of compliance may allow targeted follow-up.

## 2. Materials and Methods

### 2.1. Study Design

A retrospective cohort study was conducted using anonymized data provided by PlusDental, a brand of the Sunshine Smile GmbH (Berlin, Germany). PlusDental is a Berlin-based health-tech company, specializing in the digitalization of dental treatments and operating a digital dental care platform for aesthetic orthodontic tooth corrections, with a network of more than 200 local partner dentists. The platform integrates laboratory manufacturing of orthodontic aligners as well as treatment monitoring elements enabling dentists or orthodontists to monitor aligner changes of patients and aligner pressure using a standardized questionnaire. Among other data, this information is evaluated by the dentist or orthodontist bimonthly, and patients are provided feedback and individual instructions on wearing duration, change interval, aligner handling, or oral hygiene by e-mail or telephone. 

Our outcome was compliance of patients with regards to usage of the self-report (app-based) questionnaire and, within this, the daily aligner wearing time. Patients with consistent use of the mobile application for aligner check-in and an aligner wear time of ≥22 h on ≥75% of their aligners were classified as fully compliant. Patients with inconsistent application usage were classified as fairly or poorly compliant based on the aligner wear time: Patients with aligner wear time of ≥22 h on 50–74.9% of their aligners were classified as fairly compliant and patients with aligner wear time of ≥22 h on only <50% of their aligners as poorly compliant. The study was conducted in accordance with the World Medical Association Declaration of Helsinki and the reporting followed the STROBE checklist [20]. The data was collected as a part of the treatment and was anonymized for research use, which according to the Berlin State Hospital Act (Landeskrankenhausgesetz Berlin) and the recommendations of the Datenschutz und IT-Sicherheit im Gesundheitswesen (DIG) task force of the German Association for Medical Informatics, Biometry, and Epidemiology (GMDS) requires neither approval from an ethics committee nor informed consent. 

### 2.2. Patient Selection

A comprehensive sample of patients who finished the aligner therapy successfully with the so-called 1-1-2 CAT protocol without attachments or auxiliaries (see subsection: Orthodontic Treatment Protocol) in 2019 were included in the study. Patients were selected to conform to the following inclusion criteria: malocclusion in the anterior and premolar region to be treated with CAT, adults (>18 years) with a permanent dentition, absence of active periodontal disease, absence of local and/or systemic conditions that can affect bone metabolisms, and with no extractions required for the orthodontic treatment. These criteria coincide with the treatment scope of PlusDental (i.e., a comprehensive sample was drawn).

### 2.3. Clinical Appointment

A complete clinical examination, a full set of digital photographs, and an intraoral scan were carried out. A basic periodontal examination was performed [21,22], to rule out periodontal diseases. Added to that, patients were asked to rate their satisfaction with their current smile on a 10-point Likert scale (1 = very dissatisfied, 10 = very satisfied) and report if they had previous orthodontic treatment. 

### 2.4. Digital Treatment Planning

Virtual planning of the final desired tooth position and the required tooth movements were carried out by a dental technician using proprietary digital planning technology. The data resulting from this process was exported in the form of consecutive models. 

After the treatment plan was finalized and accepted by the dental practitioner, a 3-dimensional (3D) simulation showing the steps and the virtual final position of the teeth was sent to the patient to obtain consent on the proposed final result.

### 2.5. Manufacturing of Aligners

Additive manufacturing of the models was carried out by digital light processing technology. The aligners were embossed by a patient-specific serial number indicating the number of the step and the respective jaw to ensure ease of use by the patients. The thermoformed aligners were trimmed 2 mm above the free gingival margin.

### 2.6. Orthodontic Treatment Protocol

The treatment protocol consisted of consecutive steps of aligners which might vary according to the complexity of the case. Each aligner step was divided into three sub-steps, each with a different foil thickness. The following wear protocol was followed, 7-day wear time for the 0.5 mm thick and the 0.625 mm thick aligners, and 14-day wear time for the 0.75 mm thick aligners (1-1-2 protocol). Patients were instructed to wear each aligner for a minimum of 22 h per day, except during meals, hot drinks, and oral hygiene procedures. 

### 2.7. Treatment Follow-Up and Outcome Assessment 

**Figure 1:**
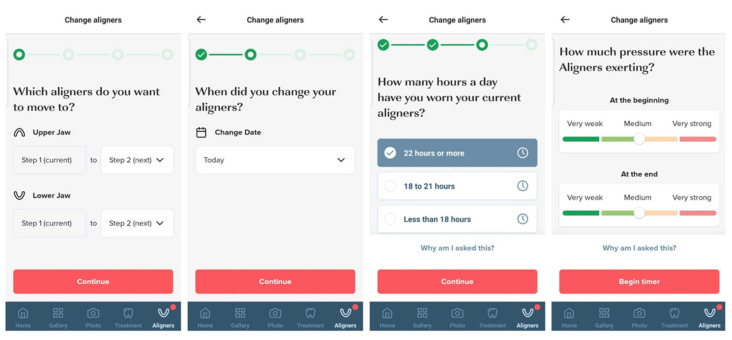
The PlusDental app-based questionnaire starting with the aligner check-in on the left.

**Figure 2:**
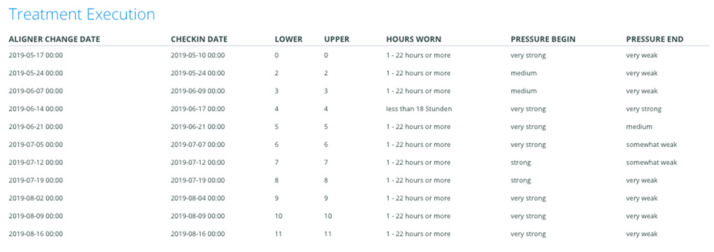
A representation of the patient treatment execution data collected through the app-based questionnaire.

The patients were instructed to check-in every aligner change using the app-based questionnaire (Figure 1 and Figure 2) and to send a set of photos every two months for follow-up through the PlusDental mobile application. The photos, aligner change date, the subjective pressure exerted at the start and the end of the aligner wear, the aligner fit, the current position of the teeth from different angles, and self-reported aligner wear duration were assessed by a dental practitioner, who then instructed the patient to continue the treatment, wear an aligner for a longer duration or repeat a step when necessary. Other comments concerning the treatment, or the oral health condition of the patient were communicated as well. At the end of the treatment process, the aligner fit, and the tooth position compared with the virtual treatment plan as well as patients’ satisfaction were assessed.

### 2.8. Statistical Analysis

Descriptive statistics were carried out and two-sided Chi-square tests were used for statistical analysis. *p*-values smaller than 0.05 were regarded as statistically significant. All calculations were conducted using JASP 0.41.1 (University of Amsterdam, Amsterdam, The Netherlands)

## 3. Results

### 3.1. Sample Characteristics

Data of all patients that finished their treatment successfully based on one intraoral scan with the 1-1-2 system in 2019 (2644 patients) was available for analysis without exclusion (comprehensive sample). Of these, 662 (25.0%) were male and 1982 (75.0%) female. The median age at treatment start was 27 years (range 18–64). When categorized by age, the largest group were young adults (18–35 years, *n* = 2223), followed by middle-aged adults (ages 36–55 years, *n* = 406). There were only a few older adults (aged older than 55 years, *n* = 15) (Table 1).

The number of aligners used per patient ranged from 6 to 36 aligners. Out of all patients, 47 (1.8%) patients were treated by 6 aligners, 434 (16.4%) patients by 9 aligners, 809 (30.6%) patients by 12 aligners, 682 (25.8%) patients by 15 aligners, 441 (16.7%) patients by 18 aligners, 150 (5.7%) patients by 21 aligners, 72 (2.7%) patients by 24 aligners, and 9 (0.3%) patients by >24 aligners.

A total of 1333/2644 (50.4%) patients reported very strong to medium pressure at the start of the aligner wear in 100% of their aligners, 345/2644 (13.0%) patients in 90 to 99.9% of their aligners, 397/2644 (15.0%) patients in 80 to 89.9% of their aligners, 193/2644 (7.3%) patients in 70 to 79.9% of their aligners, 125/2644 (7.3%) patients in 60 to 69.9% of their aligners, 100/2644 (3.8%) patients in 50 to 59.9% of their aligners, and 151/2644 (5.7%) patients in <50% of their aligners.

Most of the patients (2380/2644, 90.0%) reported medium to very weak pressure at the end of their aligner wear in comparison to the pressure exerted by the aligner at the start in 100% of their aligners, 111/2644 (4.2%) patients in 90 to 99.9% of their aligners, 95/2644 (3.6%) patients in 80 to 89.9% of their aligners, 24/2644 (0.9%) patients in 70.0 to 79.9% of their aligners, 23/2644 (0.9%) patients in 60.0 to 69.9% of their aligners, and 11/2644 (0.4%) patients in under 60% of their aligners during the treatment. Only 1 patient reported that the aligner pressure did not change over the course of each checked-in aligner.

Regarding their current smile aesthetics, 41.2% (577/1401 responders) indicated that they were “very dissatisfied” to “slightly dissatisfied” (score 1–4) while 535/1401 responders (38.2%) were “neutral” (score 5–6), and 20.6% (289/1401 responders) were “slightly satisfied” to “very satisfied” (score 7–10).

A total of 1038/2644 (39.3%) patients reported previous orthodontic treatment, 702/2644 (26.6%) reported no previous orthodontic treatment, and 904/2644 (34.2%) could not answer the question. The patients who indicated previous orthodontic treatment had removable appliances in 420/1038 (40.5%) cases, fixed appliances in 501/1038 (48.3%) cases, and both removable and fixed appliances in 117/1038 (11.3%) of the cases.

Patients who had reported a previous orthodontic treatment answered in 847 (81.6%) of the cases that they did not have any retainer anymore, 98 (9.4%) had a fixed retainer, 87 (8.4%) had removable retainers, and 6 (0.6%) had both removable and fixed retainers.

### 3.2. Compliance

Patients were classified according to the compliance criteria into full, fair, and poor compliance. A total of 953/2644 (36.0%) of patients showed full compliance, 1012/2644 (38.3%) fair compliance, and 679/2644 (25.7%) poor compliance. 

A total of 1203/2644 (45.5%) patients wore each aligner for 22 h per day throughout the treatment period, 456/2644 (17.2%) patients deviated in 0.1–25% of their aligners, 306/2644 (11.6%) patients deviated in 25.1–50% of their aligners, 211/2644 (8.0%) patients deviated in 50.1–75% of their aligners, and 468/2644 (17.7%) patients deviated in >75% of their aligners.

Compliance was higher in males compared with females (*p* < 0.05). No significant differences were found for patient group age in patient compliance (*p* = 0.097) (Table 2).

There were no significant differences in patient compliance when treatments stretched over different time periods (*p* = 0.268) (Table 3).

Compliance was not different for patients with different smile aesthetics satisfaction before treatment (*p* = 0.110) (Table 4).

A significant difference in patient compliance was found for patients regarding previous orthodontic treatment. Patients without previous orthodontic treatment showed better patient compliance (*p* = 0.023) (Table 5).

Further analysis of patients with previous orthodontic treatment classified according to the type of previous treatment showed that patients treated only with removable appliances were shown to be the most compliant (*p* = 0.0472) (Table 6).

## 4. Discussion

CAT is increasingly popular for orthodontic corrections but relies heavily on patients’ compliance. The present study evaluated the compliance and compliance-associated factors in 2644 CAT patients in 2019. Based on our findings, males were slightly more likely than females to be compliant while age and pre-treatment satisfaction with one’s own smile was not associated with compliance. Most significantly, individuals who had previous orthodontic treatment showed significantly lower compliance. To the knowledge of the authors, this is the first study evaluating compliance of a large cohort of clear aligner patients using a mobile application during a remote follow-up in terms of mobile application usage and self-reported wearing hours.

Our findings require some detailed discussion. Gender has been found to be associated with compliance for orthodontic treatment before, while the direction of any association remains unclear. For example, Al-Abdallah et al. found female patients to be more compliant during fixed orthodontic treatment [16], Schäfer et al. found female patients to be more compliant during removable orthodontic treatment [23], while Crouse found no difference between males and females [24]. The patients in these studies were either all below 18 years of age [16,23], or partially under 18 [24], which could be the reason for the opposite results found in our study where the youngest patients at the start of the treatment were 18 years old (median = 27). It is possible that in adults, men are more compliant during orthodontic treatment. This finding, however, needs to be further confirmed in a sample with a bigger age range including adolescents and children, and should be explored using qualitative research methods as well to better understand reasons behind it.

Similarly, age has been ambiguously found to be associated with compliance. For example, Barbosa et al. showed that adult patients were more compliant with fixed appliance therapy than adolescents [17], whereas Crouse found that patients in the 14–19 and 20–39 age groups were significantly less compliant with CAT than those younger or older. The absence of an adolescent age group in the current study could be the reason why such effect of age on compliance could not be established, together with the small number of patients (15/2644) in the oldest age group which was expected to be less compliant using the mobile application. Overall, the comparison of age groups likely suffered from limited heterogeneity and hence statistical power, as most patients were of similar age.

The absence of a demonstrable effect of satisfaction with the current smile before treatment and compliance during the treatment is in line with the findings of Mandall et al. [18], where concern about the negative impact of teeth appearance was not indicative of higher compliance during the treatment. This is somewhat counterintuitive, as one may expect individuals unhappy with their smile to desire an aesthetic improvement more eagerly than those less unsatisfied, who in turn may be less compliant. It is possible, however, that all adult patients in our sample where generally relatively interested in improving their smile aesthetics, mainly demonstrated by them paying out of pocket for a by-large aesthetic correction.

Notably, patients who had experienced previous orthodontic treatment were shown to be less compliant during CAT. A possible explanation is that those patients might have been non-compliant previously, for example with their retention protocol, leading to relapse in the first place (81.6% of the patients who had previous orthodontic treatment reported not having retainers at the time of their clinical appointment). A significant decrease in compliance over time regarding daily retainer wear and/or the wearing hours is a common finding among orthodontic patients [25], especially for relatively long orthodontic treatment processes [19]. Individuals who had experienced previous therapy may perceive the second therapy as especially long and hence become impatient earlier, impacting on compliance. 

It is worth noting that the patients who had previous orthodontic treatment with removable appliance were found to be more compliant than the patients who had previous fixed orthodontic treatment. The familiarity with the removable appliances and their mode of action might have been the reason behind the higher compliance of patients with previous removable appliance experience during CAT.

Based on the findings in this study, the dental practitioner might be able to identify potential low compliance CAT patients which would allow for early intervention to try to improve compliance during remote follow-up. Among many methods, praising the patient for compliant behavior and patient education about the consequences of poor compliance were reported by orthodontists to be of high importance in improving compliance [26]. In a remote follow-up CAT context, that could translate into delivering praise to patients with compliant wear time and consistent application usage and educating the patient about the consequences of poor compliance prior to treatment and sending them reminders during treatment when deviation from compliant behavior is observed. The efficacy of these methods needs to be further studied.

This study comes with a range of limitations. First, the absence of an under 18 years old (children and adolescents) age group together with the small number of patients in the oldest age group (above 56 years old) did not allow for a comprehensive representation of the orthodontic treatment-seeking population, especially those below the age of 18 years. Second, the aligner wearing hours and the perceived aligner pressure were recorded using self-reports, which are susceptible to biases such as overstatement and understatement or distortion of perception and memory [27]. This is why we classified the patients in this study based on the consistency of mobile application usage during remote follow-up as a more objective indicator of compliance. Third, compliance was measured for CAT; inferences on other orthodontic therapies should not be made. Last, the sample suffered from selection bias; only patients willing to pay a certain amount of money out-of-pocket from mainly three countries, Germany, Switzerland, and Austria, for one specific aligner therapy were included. Generalization to other populations should not be attempted. 

Further studies are needed to evaluate the influence of other factors on compliance such as the type of malocclusion, the aligner thickness, the frequency of aligner breakage, and the need for IPR.

## 5. Conclusions

Within the limitations of this study, gender but not age or pre-treatment satisfaction with one own’s smile was associated with patient compliance during CAT. Most notable, patients who had experienced previous orthodontic therapy showed significantly lower compliance. Based on our findings, individuals at-risk should be identified in future studies and their compliance prospectively recorded. Our findings could be used to guide practitioners towards limitedly compliant individuals, allowing early intervention.

## Figures and Tables

**Table 1:** Age group and gender distribution of the overall sample.

	18- to 35- Years Old	36- to 55- Years Old	56- to 64- Years Old	Total
**Male**	563 (21.2%)	96 (3.6%)	3 (0.1%)	662 (25.0%)
**Female**	1660 (62.7%)	310 (11.7%)	12 (0.4%)	1982 (75.0%)
**Total**	2223 (84%)	406 (15.3%)	15 (0.5%)	2644 (100%)

**Table 2:** Compliance in different age groups and by gender.

	Overall Sample	Full Compliance	Fair Compliance	Poor Compliance	Chi-Square
**Gender *n* (%)**					
**Male**	662 (25%)	261 (9.8%)	277 (10.5%)	124 (4.7%)	X2 (2, *n* = 2644) = 22.34 *p* = 0.000014 (*p* < 0.05)
**Female**	1982 (74.9%)	692 (26.1%)	735 (27.8%)	555 (21.0%)	
**Age group *n* (%)**					
**18- to 35- years old**	2223 (84%)	794 (30%)	852 (32.2%)	577 (21.8%)	X2 (4, *n* = 2644) = 7.84 *p* = 0.097
**36- to 55- years old**	406 (15.3%)	156 (5.9%)	156 (5.9%)	94 (3.6%)	
**56- to 64 years old**	15 (0.5%)	3 (0.1%)	4 (0.2%)	8 (0.3%)	
**Total**	2644 (100%)	953 (36%)	1012 (38.3%)	679 (25.7%)	

**Table 3:** Comparisons of compliance by treatment duration.

	Overall Sample	Full Compliance	Fair Compliance	Poor Compliance	Chi-Square
	**Treatment duration *n* (%)**				
**2 months**	47 (1.7%)	20 (0.8%)	14 (0.5%)	13 (0.5%)	X2 (18, *n* = 2644) = 21.22 *p* = 0.268
**3 months**	434 (16.4%)	143 (5.4%)	165 (6.2%)	126 (4.8%)	
**4 months**	809 (30.5%)	308 (11.6%)	285 (10.8%)	216 (8.2%)	
**5 months**	682 (25.7%)	244 (9.2%)	281 (10.6%)	157 (5.9%)	
**6 months**	441 (16.6%)	147 (5.6%)	184 (7.0%)	110 (4.2%)	
**7 months**	150 (5.6%)	60 (2.3%)	53 (2.0%)	37 (1.4%)	
**8 months**	72 (2.7%)	27 (1.0%)	28 (1.1%)	17 (0.6%)	
**9 months**	7 (0.2%)	4 (0.2%)	1 (0.0%)	2 (0.1%)	
**10 months**	1 (0.03%)	0 (0.0%)	0 (0.0%)	1 (0.03%)	
**12 months**	1 (0.03%)	0 (0.0%)	1 (0.03%)	0 (0.0%)	
**Total**	2644 (100%)	953 (36%)	1012 (38.3%)	679 (25.7%)	

**Table 4:** Patient compliance and smile aesthetics satisfaction.

	Total Responders	Full Compliance	Fair Compliance	Poor Compliance		Chi-Square
**Smile aesthetics satisfaction *n* (%)**						
**Very satisfied to** **Slightly satisfied**	289 (20.6%)	96 (6.9%)	119 (7.0%)	74 (4.4%)	X2 (4, *n* = 1401) = 7.54 *p* = 0.110	
**Neutral**	535 (38.2%)	184 (13.1%)	201 (11.8%)	150 (8.8%)		
**Slightly dissatisfied to** **Very dissatisfied**	577 (41.2%)	217 (15.5%)	237 (13.9%)	123 (7.2%)		
**Total**	1401 (100%)	497 (35.5%)	557 (32.7%)	347 (20.4%)		

**Table 5:** Patient compliance and previous orthodontic treatment.

	Total Responders	Full Compliance	Fair Compliance	Poor Compliance	Chi-Square
**Previous orthodontic treatment *n* (%)**					
**Yes**	1038 (59.7%)	357 (20.5%)	391 (22.5%)	290 (16.7%)	X2 (2, *n* = 1740) = 7.49 *p* = 0.023 (*p* < 0.05)
**No**	702 (40.3%)	252 (14.5%)	294 (16.9%)	156 (9.0%)	
**Total**	1740 (100%)	609 (35%)	685 (39.3%)	446 (25.6%)	

**Table 6:** Patient compliance and type of previous orthodontic treatment.

	Total Responders	Full Compliance	Fair Compliance	Poor Compliance		Chi-Square
**Type of p** **revious orthodontic treatment *n* (%)**						
**Removable appliance**	420 (40.5%)	158 (15.2%)	165 (15.9%)	97 (9.3%)	X2 (4, *n* = 1038) = 9.62 *p* = 0.0472 (*p* < 0.05)	
**Fixed appliance**	501 (48.3%)	159 (15.3%)	181 (17.4%)	161 (15.5%)		
**Both removable & fixed**	117 (11.3%)	40 (3.9%)	45 (4.3%)	32 (3.1%)		
**Total**	1038 (100%)	357 (34.4%)	391 (37.7%)	290 (27.9%)		

## Data Availability

Data available on request due to privacy restrictions.
